# A study on the influencing factors of rural land transfer willingness in different terrain areas——Based on the questionnaire survey data of Anhui Province and Qinghai Province, China

**DOI:** 10.1371/journal.pone.0303078

**Published:** 2024-06-07

**Authors:** Ershen Zhang, Guoen Wang, Yuwei Su, Guojun Chen

**Affiliations:** School of Urban Design, Wuhan University, Wuhan, Hubei Province, China; East China Normal University, CHINA

## Abstract

This study delves into the factors influencing the willingness of rural land transfers in different terrain areas, aiming to promote the improvement of land transfer institutions and accelerate the process of scale farming. Based on rural survey data from Anhui and Qinghai provinces in China, this research uses geographical detector and Binary Logistic Model to explore the differential factors affecting the willingness of farmers to participate in land contract transfer in the first and third terrain areas of China. The study examines four dimensions, including individual characteristics, family endowments, social support strategies, and geographical environment. The findings reveal the following: (1) By comparing the mean values, standard deviations, and coefficients of variation of the data from both provinces, it is evident that the indicators of individual characteristics, family endowments, social support strategies, and geographical environment differ significantly between the two provinces. This indicates substantial disparities in the basic attributes of farmers and their living environments. (2) The single-factor explanatory power significantly influencing farmers’ willingness to engage in land transfer varies considerably and is statistically significant at the 1% level. The types of interaction between two factors mainly include dual-factor enhancement, nonlinear enhancement, single-factor nonlinear attenuation, and nonlinear attenuation. (3) There are commonalities and differences in the factors that significantly influence farmers’ willingness to participate in land transfer in the two provinces. Common factors influencing farmers’ land transfer willingness in both provinces include: the educational level of household heads, the health status of household heads, the number of family laborers, the arable land area, the differentiation of agricultural management objectives, the proportion of agricultural operating income, labor service economy, and relocation policies. Factors showing different influences include: the age of household heads, school-age children, the number of family members engaged in different occupations, the proportion of income from off-farm employment, minimum guarantee policies credit support, location distance, and terrain undulation. Therefore, in formulating land transfer policies, the government should prioritize significant driving factors influencing farmers’ decision-making behavior in different regions. It is essential to develop and implement land transfer policies tailored to local conditions with the primary goal of safeguarding the rights and interests of the principal stakeholders, thus achieving sustainable land utilization.

## 1. Introduction

Land transfer is a product of rural economic development reaching a certain stage. The large-scale transfer of idle land can provide basic conditions for the implementation of large-scale, intensive and modern agricultural production mode [1–3]. Due to the different historical backgrounds of the establishment of capitalist systems in various countries, the forms of land ownership differ to some extent. Representative examples include China, the United Kingdom, and the United States [4–7]. The enclosure movement in the United Kingdom during the 16th to 19th centuries eliminated the small peasant class and created proletarians who could freely sell their labor. This led to the establishment of the typical “landowner-tenant farmer-wage laborer” trinity, forming the capitalist farm system [8–11]. Land transfer in the United States began with the colonial wars and acquisitions in the late 18th century. During the establishment and development of family farms, land leasing and tenancy were used to expand the scale. The US government retained only legal ownership and corresponding income rights to publicly owned land, while all other rights belonged to the family-operated entities. This created necessary opportunities and impetus for the release of the inherent potential of the rural land management system [12, 13]. The transformation of China’s rural land system, which can be traced back to 1978, has led to a complex evolution of the land rights structure. This transformation was driven by the country’s changing development needs, gradual improvements in land efficiency, relaxation of property rights regulations, and the gradual return of property rights to farmers [14, 15]. In the current period, land transfer in rural China refers to the transfer of operational and usage rights while farmers retain their land contract management rights [5, 16, 17].

Since 2004, the Chinese government’s Central Document No. 1 has consistently emphasized the need to deepen rural land system reforms for 20 consecutive years, gradually institutionalize and scale up land transfer in rural areas [18, 19]. In 2019, Chinese government issued the “Opinions on Maintaining the Stability and Permanence of Land Contracting Relationships”, proposing to activate land management rights, safeguard contracting rights, promote the separation of “three rights” (ownership, contract and management rights) in rural land, and develop various forms of moderate-scale agricultural operations [20–22]. In 2021, the Ministry of Agriculture and Rural Affairs of China issued the “Management Measures for Rural Land Transfer of Operational Rights”, further clarifying the rights, obligations, and responsibilities of both parties involved in the transfer process, and promoting the orderly transfer of land management rights [23]. Promoting moderate-scale farming in agricultural development is of great significance as it allows for orderly land transfer while fully respecting the status of all parties involved. These policy measures highlight the fact that there is still an incomplete satisfaction of the willingness for land transfer in China’s agricultural areas. Various factors influence the willingness of farmers to engage in land transfer, and the mechanisms involved are complex. Therefore, analyzing the commonalities and differences of factors influencing the willingness of rural land transfers in China will not only expedite the process of scaling and intensifying land use in rural areas, optimizing land resource allocation, but also facilitate agricultural structural adjustments and accelerate the process of agricultural industrialization. Additionally, it will promote rural economic development, labor force migration, attract social capital investment in agricultural production and development, and effectively increase farmers’ income.

In the study of land transfer behavior, some scholars have adopted a macro perspective, analyzing the current situation and influencing factors of land management rights transfer at the national, provincial, municipal, and county levels [24–26]. However, there is a lack of comparative analysis among different case regions. Other scholars have taken a micro perspective, exploring the internal factors of farmers’ land transfer behavior by analyzing factors such as household composition [27, 28], characteristics of the household head [29], livelihood strategies [30], economic income structure [31], social assistance policies [32, 33], and attachment to land [34]. External factors influencing farmers’ participation in land management rights transfer include the market situation of land transfer [35], government behavior [36], natural geographic environment [37], location characteristics [38], land scale [39], and land quality [5, 40]. Additionally, during the transfer process, rental income obtained from participation in land transfer can serve as a channel for household income expansion, but the benefits also vary greatly [41, 42]. Existing research on the factors influencing land transfer willingness has categorized the outcomes of land transfer willingness into transfer and non-transfer [5, 43, 44]. Scholars have used various methods such as Logistic model, SBM-DEA model, Probit model, Tobit model, etc., to study farmers’ land transfer behavior [24, 39, 45, 46]. However, the use of a single method alone cannot reflect the relationship between factors or the combined effects of bivariate variables on land transfer willingness. Moreover, the construction of indicator systems often relies on questionnaire data and lacks a comprehensive analysis of panel data, natural topography data, and assistance policies. Therefore, employing multiple methods to comprehensively analyze data from various sources can enhance the rigor of research conclusions.

Based on the review of research on land management rights transfer behavior across multiple fields, it is found that farmers in rural areas of China are mainly influenced by factors such as individual characteristics of household members, household endowments, socio-economic factors, government policies, and natural geographical conditions. However, there is a lack of comparative analysis of the factors influencing farmers’ land transfer willingness in different topographic regions, which hinders the implementation of government land transfer policies tailored to local conditions and individual households. China’s topography exhibits distinct three-level features, with the first level centered around the Qinghai-Tibet Plateau, which has an average elevation of over 4,000 meters. The third-level topographic features include vast plains in eastern China with elevations below 200 meters and hilly regions in southern China where elevations generally do not exceed 500 meters. Residents living in different topographic levels exhibit significant differences in their production and daily activities. Therefore, in this study, Qinghai Province and Anhui Province were selected as research areas. Based on field survey data, government statistics, and terrain data, the study employed Geographical Detector and Binary Logistic Model to explore the common and differential factors influencing farmers’ willingness to transfer land management rights in different topographic regions from four dimensions: household individual characteristics, household endowments, social assistance strategies, and geographical environment. The study aims to provide targeted policy recommendations. The research findings are expected to provide reference for government departments in improving risk prevention and guarantee mechanisms for land management rights transfer, promoting smooth land transfer activities, achieving agricultural scale management, and contributing to the harmonious, stable, and revitalized development of rural areas.

## 2. Materials and methods

### 2.1. Overview and typical characteristics of the study area

Qinghai Province is situated in the northeastern part of the Qinghai-Tibet Plateau, known as the “Roof of the World”. It spans an area of 722,300 square kilometers and lies within the first step of China’s terrain. The province exhibits significant variations in terrain, sloping from west to east. The maximum elevation difference within the province reaches 5,210 meters. The dominant landform in this area is mountainous, with some plains and hills. It is categorized as part of the western economic zone and experiences a plateau continental climate. In 2022, the rural population was approximately 2.29 million. The urbanization rate of permanent residents was 61.43%. The average per capita disposable income for rural residents was 14,456 yuan (CNY, Chinese Yuan). The primary industry contributes to 10.5% of the overall economic output, and the total area of land used for grain cultivation throughout the year is 303,470 hectares, primarily focused on wheat, barley, and corn.

Anhui Province is located in the eastern region of China, covering an area of 139,600 square kilometers. It lies within the third step of China’s terrain and generally exhibits a west-high-east-low and north-high-south-low trend. The province comprises various landforms, including plains, hills, and mountains. It is situated in the central and eastern economic zone and lies within the transitional region between the warm temperate and subtropical climates. In 2022, the rural population was approximately 24.4 million, with an urbanization rate of 60.2%. The average per capita disposable income for rural residents was 19,575 yuan. The primary industry contributes to 7.8% of the overall economic output, and the total area of land used for grain cultivation throughout the year is 7,314,267 hectares, primarily focused on rice, wheat, and corn.

**Fig 1:**
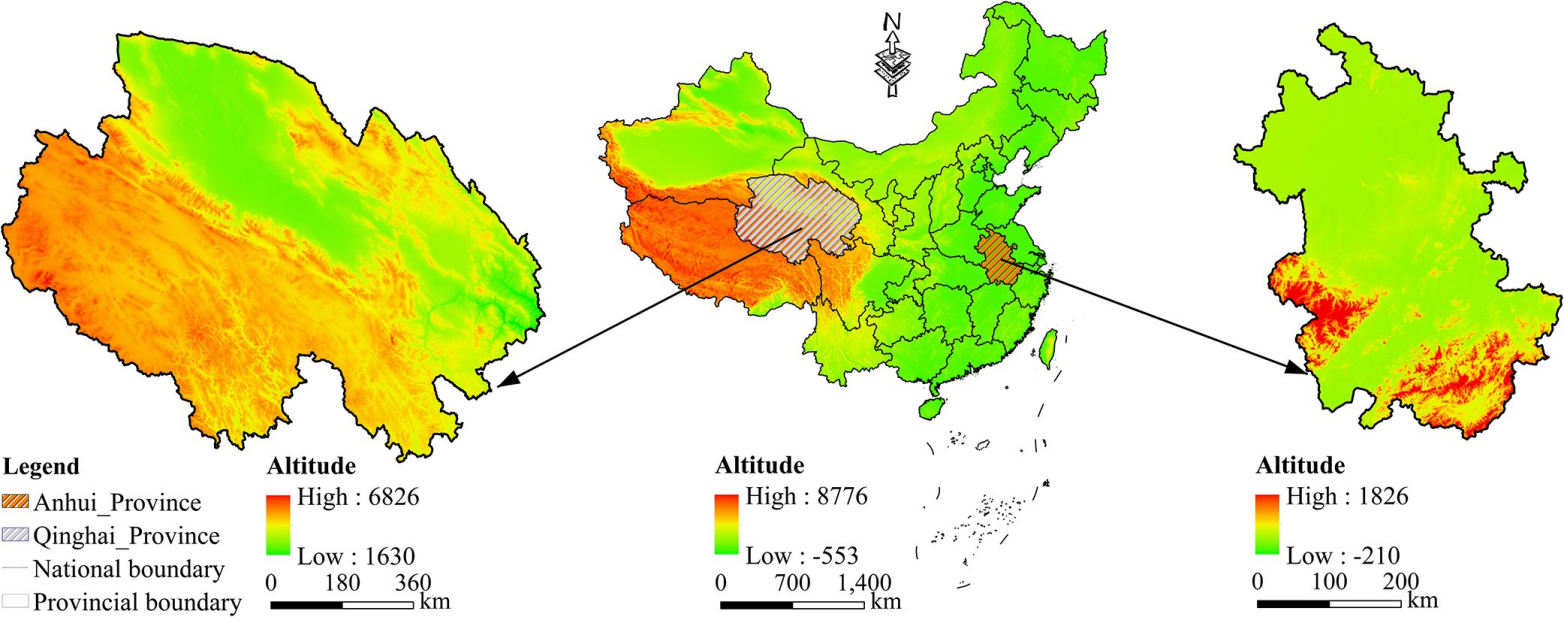
Overview map of Qinghai Province and Anhui Province (map review number GS [2020] 4619). (https://www.tianditu.gov.cn/).

China has vast rural areas, all facing opportunities and challenges brought about by the policy of transferring land management rights. This study primarily focuses on rural families in two provinces. The surveyed families are all located in rural areas and were primarily engaged in agricultural production before the promulgation of the “Rural Land Contract Law of the People’s Republic of China” in 2002. Despite significant environmental differences in different topographical areas, they all possess relatively abundant natural resources, including cultivated land, grasslands, and mountainous areas [47–50]. However, with the severe loss of rural labor force, the utilization of existing cultivated land significantly affects the livelihoods of households and the local economic development. Contrasting the regional attributes of Qinghai Province and Anhui Province (Table 1), they exhibit the following typical differences: (1) Natural topographic differences: Anhui Province is located at the third-tier level in China, characterized by diverse terrain types, while Qinghai Province is situated at the first-tier level, predominantly composed of plateaus and mountains (Fig 1). (2) Climate features differences: Anhui Province exhibits a significant monsoon climate with distinct seasons, concentrated summer rainfall, pleasant autumn weather, and cold winters. Qinghai Province, on the other hand, is characterized by low temperatures, large diurnal temperature variations, and intense radiation. (3) Disparities in regional economic conditions: In 2022, Anhui Province’s annual GDP reached 4.5045 trillion yuan, which is 12.48 times higher than Qinghai Province’s GDP. The per capita disposable income of rural residents in Anhui Province is1.35 times as high as that of Qinghai Province. (4) In 2022, Anhui Province ranked fourth in grain production in China, while Qinghai Province ranked 27th. We use the Hu Huanyong Line as a boundary and simultaneously consider the geographical differences of The three steps of China’s terrain [51]. Representative provinces from the western and eastern regions are selected as sample areas. This approach allows for the reflection of the typical characteristics of the sample areas and creates strong contrasts in the environmental conditions contributing to scientific phenomena.

**Table 1:** Comparison of study area profiles.

Study Area Profile	Qinghai Province	Anhui Province
Area	722,300 square kilometers	139,600 square kilometers
Topography	First step of China’s terrain	Third step of China’s terrain
Climate	Plateau continental climate	Monsoon climate
Rural population	2.29 million	24.4 million
Urbanization rate	61.43%	60.2%
Rural per capita disposable income (yuan/CNY)	14,456	19,575
Proportion of the primary industry’s output value	10.5%	7.8%

### 2.2. Research data

The research data primarily came from the following sources: (1) During the field research, considering the varying development situations in each township, the research team adopted a method of stratified cluster sampling to select samples from villages. Firstly, for each selected case area in each province, villages were sorted based on the population registered in household registers. Secondly, a random equidistant method was used to select sample villages. This method helps mitigate the impact of uneven development within the region and ensures representation of villages and households with diverse development levels, enhancing the sample’s representativeness. In addition, interviews were conducted with a proportion of 30% of the registered population in each village. Finally, the interview results were cross-verified with the data provided by the village committees. The questionnaire content mainly included basic information about household members, production and consumption information, and social assistance measures. In Anhui Province, a total of 740 questionnaires were obtained, excluding those with errors or damage, resulting in 712 valid questionnaires and an effective rate of 96.22%. In Qinghai Province, a total of 540 household questionnaires were obtained, with 515 valid questionnaires and an effective rate of 95.38%. (2) Basic data on the socio-economic and geographical confidence of Qinghai Province and Anhui Province were primarily obtained by consulting the statistical yearbooks and government bulletins at the provincial, municipal, and county levels (Table 1). (3) Geospatial data were obtained from the Geospatial Data Cloud Platform and the Resource and National Platform for Common Geospatlal Informatlon Services (https://www.tianditu.gov.cn/). Data including administrative divisions, DEM elevation, and road traffic were processed using ArcGIS software.

### 2.3. Research methods

**Fig 2:**
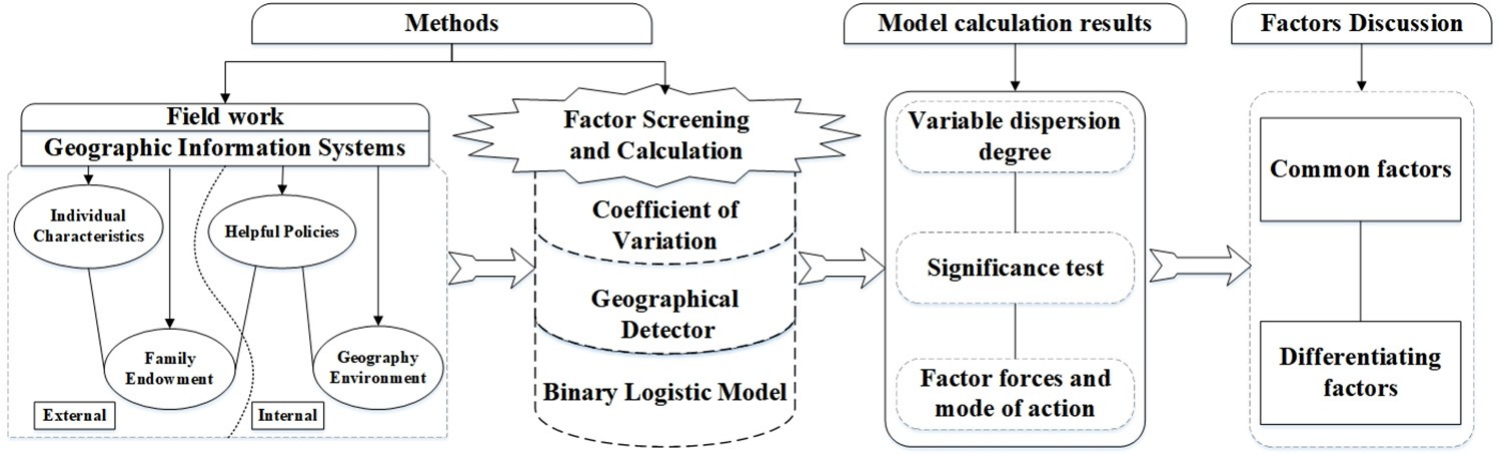
Methods workflow.

When facing changes, farmers adjust land use to suit their families’ overall situation. Continuous land reorganization in rural areas adapts to evolving societal needs and involves formulating land utilization and management systems by governments and local organizations. Hence, rural farmers’ land transfer behavior closely correlates with government policies on land use formulation and implementation. Exploring factors affecting farmers’ land transfer willingness with scientific methods is crucial and reliable. Therefore, this study developed scientific research methods based on a comprehensive review of the literature (Fig 2). The research methods in this paper can be divided into three main parts:

1. The data processing function of ArcGIS software is used to extract natural geographical elements and location factors from vector data, enriching the research database.
2. The coefficient of variation of each index factor is calculated by calculating the standard deviation and mean, conducting comparative analysis of the dispersion degree and average level of each factor.
3. By comparing the advantages of various measurement methods in data management, statistical analysis, factor analysis, and output management in software such as Excel and SPSS, Geographical Detector and Binary Logistic Model are finally selected to statistically and standardize the data. This systematically explores the common and differential factors influencing the willingness of farmers in Anhui Province and Qinghai Province to transfer land, and determines the magnitude and mode of the driving forcesof each factor.

#### 2.3.1. Fieldwork method

The fieldwork method, also known as on-site investigation or field research, falls under the scope of anthropology. This method is widely used in research in both natural and social sciences [52–54]. In this study, the investigators first lived in the research area for a period of time. After familiarizing themselves with the local society, culture, and other aspects, they recorded information about the respondents’ family members, land resources, and economic income through questionnaire interviews. Unlike traditional fieldwork methods, this study focuses on scientific questions primarily related to objective facts provided by the respondents. Therefore, after the on-site investigation, the required data for the study were obtained by analyzing the questionnaire results.

#### 2.3.2. GIS technology

Using ArcGIS software’s data processing capabilities for multi-source geographical information [55–57], the location and topographic conditions of the farmers’ residences are analyzed, and geographic attribute data are extracted. Based on the farmers’ spatial attribute data and China’s digital elevation model (DEM) data, the following steps are taken:

1. Using the DEM data of the administrative region to which the farmers belong, the “Spatial Analyst” function analysis module is employed along with the “Neighborhood analysis” and “Focal statistics” tools to extract the maximum and minimum values of the DEM data. Then, the “Raster calculator” is used to calculate the difference between the maximum and minimum DEM values, resulting in the terrain ruggedness.
2. Based on the farmers’ spatial attribute data and vector data of road networks, the distance to the nearest city is calculated.

#### 2.3.3. Coefficient of variation

Considering that the two sets of data in this study have different units of measurement, directly comparing their dispersion using the standard deviation may yield inadequate results. The coefficient of variation (Coefficient of Variation) is obtained by dividing the standard deviation by the mean of the data. It is a normalized measure of the degree of dispersion in a probability distribution, reflecting the absolute value of data dispersion, and it can also eliminate the issue of different units of measurement [58–61]. Therefore, this method was used in this study to calculate the coefficient of variation to mitigate the effects of inconsistencies in the magnitudes and to facilitate comparative analysis of the dispersion of the independent variables in the two acoustic sub-data sets. The formula for calculating the coefficient of variation is as follows:

$$c_{v}=\frac{\sigma }{\mu }$$ (1)

where ***C***_***v***_ reflects the degree of dispersion and the magnitude of the average level of the variable, ***σ*** represents the standard deviation of the data set, and ***μ*** represents the mean of the data.

#### 2.3.4. Geographical detector

Geographical detector, widely used in various fields of research, offers several advantages, including immunity to multicollinearity, avoidance of causal relationships between independent and dependent variables, and the ability to explore the joint effects of two factors on the dependent variable [62–66]. In this study, leveraging the advantages of this method, a preliminary screening of 26 indicators was conducted to identify the factors that significantly influence farmers’ willingness to transfer land and to construct the final indicator system. Following the treatment of independent variable ***X*** and dependent variable ***Y*** in relevant studies, the single-factor detection module and the interaction detection module of this method were used to analyze the significance of the independent variable ***x***_**i**_ in the indicator system. This analysis helped determine the significant and explanatory factors of farmers’ willingness to participate in land transfer. The calculation formula is as follows:

$$q=1-\frac{\sum_{h=1}^{L}N_{h}\sigma_{h}^{2}}{{N\sigma }^{2}}$$ (2)

where ***q*** represents the explanatory power of the driving factor, indicating the degree to which the influencing factor ***X***_***i***_ explains the variation in the quantitative measurement indicator ***Y*** of land transfer willingness. A larger value indicates a stronger influence. ***L*** represents the number of layers of variable factors. ***N*** and ***N***_***h***_ represent the total number of farmer samples and the number of samples within layer ***h***, respectively. ***σ***_***h***_^***2***^ and ***σ***^***2***^ represent the variance of the overall study area and the variance within layer ***h***, respectively.

#### 2.3.5. Binary logistic model

Binary logistic models are widely used in the study of scientific issues such as land transfer, sustainable use, and trading [67–69]. Dividing the dependent variable reveals that it is binary (0 or 1), aligning with the requirements of the binary Logistic regression model. Therefore, this study chooses the binary Logistic regression model to analyze the influencing factors of farmers’ willingness to transfer land management rights, aiming to identify the relationship between the independent variables and the dependent variable. The specific form of the model is as follows:

$$\ln\frac{P}{1-P}=b_{0}+b_{1}x_{1}+\cdots +b_{k}x_{k}$$ (3)

In the equation: b0 represents the constant term, indicating the natural logarithm of the odds when all independent variables are zero; parameters ***b***_**1**_, ***b***_**2**_,…, ***b***_**k**_ are estimated parameters for the variables affecting land transfer willingness. They signify the change in the natural logarithm of the odds (*OR*) when the value of the respective independent variable increases by one unit, keeping all other independent variables constant; ***P*** represents the probability of the event ***Y*** occurring, with a range between 0 and 1, while 1-***P*** is the probability of the event not occurring. By analyzing the estimated values of the parameters ***b***_**i**_ (except for b_1_) and other empirical results from the logistic regression, the effects of various influencing factors can be examined.

## 3. Results

### 3.1. Variable Selection and Explanation

The engagement of rural households in land transfer activities is the result of the combined influence of various factors. To build the necessary database for this study, relevant data on household situations, social factors, natural environment, etc., were collected. Drawing on the research findings of scholars on land transfer issues [24–29, 31–33, 37, 38] and utilizing geographical and economic methods, in conjunction with the basic conditions of the study cases, 32 indicator data related to farmers’ willingness to engage in land transfer were subjected to collinearity tests. After excluding 15 non-significant and collinear indicators, 17 variables that significantly impact farmers’ participation in land transfer activities in the study cases were selected to construct the analytical framework for influencing factors in this study (Table 2). These factors were categorized into four classes: household head characteristics, endowment situation, social support strategies, and geographical environment. The combination of geographic detectors and binary Logistic regression models was chosen, leveraging the advantages of both methods in data processing and enabling mutual validation of experimental results. Initially, the mean, standard deviation, and coefficient of variation for the 17 variables in two sets of dimensional data for Anhui Province and Qinghai Province were calculated for preliminary analysis.

**Table 2:** Variables. (https://github.com/zhangershen/DATE.git).

Endowment Type	Variabl	Variable Assignment	Anhui Province Surveyed Households			Qinghai Province Surveyed Households		
			Mean	Standard Deviation	Coefficient of Variation	Mean	Standard Deviation	Coefficient of Variation
Individual characteristics of the household head	Age (*x*_1_)	Actual Measurements:	64.43	13.58	0.21	51.40	11.83	0.23
	Education Level (*x*_2_)	1- Illiterate/Semi-illiterate; 2- Primary School; 3- Junior High School; 4- High School/Technical School; 5- College/Undergraduate and above	1.92	0.84	0.44	2.12	0.57	0.27
	Health Level (*x*_3_)	1- Disease or disability; 2- Healthy	1.29	0.45	0.35	1.44	0.50	0.35
Family endowment situation	Number of Household Labor Force (*x*_4_)	Number of family members with labor capacity (age between 18 and 60, excluding students)	1.32	1.33	1.01	1.95	1.04	0.53
	School-Age Children (*x*_5_)	0- No; 1- Yes	0.32	0.47	1.47	0.40	0.49	1.23
	Cultivated Land Area (*x*_6_)	Actual area of cultivated land (hectares)	5.41	4.04	0.75	5.31	4.21	0.79
	Diversification of Cultivated Land Management Objectives (*x*_7_)	1- To meet family grain consumption; 2- To meet grain consumption while increasing income; 3- Solely for increasing income	2.28	0.48	0.21	2.39	0.52	0.22
	Number of Job Categories (*x*_8_)	Number of family members engaged in sideline activities	0.99	0.98	0.99	1.34	0.77	0.57
	Proportion of Agricultural Operating Income (*x*_9_)	Annual agricultural operating income/family annual total income	0.20	0.21	1.05	0.20	0.21	1.05
	Proportion of Wage Income from Employment (*x*_10_)	Annual wage income/family annual total income	0.38	0.34	0.89	0.50	0.28	0.56
	Per Capita Disposable Income (*x*_11_)	1- Low income (<10,000 RMB); 2- Medium income (10,000–50,000 RMB); 3- High income (>50,000 RMB)	1.52	0.50	0.33	2.38	0.51	0.21
Social assistance strategies	Minimum Guarantee Policy (*x*_12_)	Benefiting from national minimum living security/five-guarantee policies: 0- No; 1- Yes	0.50	0.50	1.00	0.44	0.50	1.14
	Credit Support (*x*_13_)	Whether obtaining credit support: 0- No; 1- Yes	0.45	0.50	1.11	0.06	0.24	4.00
	Labor Service Economy (*x*_14_)	Engaging in labor services economy development: 0- No; 1- Yes	0.21	0.41	1.95	0.17	0.38	2.24
	Relocation policy (*x*_15_)	Undergoing relocation: 0- No; 1- Yes	0.05	0.22	4.4	0.20	0.40	2
Geographic environment	Location Distance (*x*_16_)	Actual distance from residence to the nearest county town (km)	16.24	7.26	0.45	21.75	15.84	0.73
	Terrain Fluctuation (*x*_17_)	Altitude range within the county area (km)	124.31	82.02	0.66	159.54	144.50	0.91

In terms of household head characteristics, there are significant differences in the educational level of interviewed farmers between Qinghai Province and Anhui Province. The coefficient of variation for the educational level indicator is noticeably larger in Anhui Province than in Qinghai Province, indicating a more evenly distributed educational level among interviewed farmers in Anhui Province.

In terms of household endowment situation, there are significant differences between interviewed farmers in Anhui Province and Qinghai Province in terms of the number of household labor force, the number of individuals involved in occupational differentiation within the household, and the level of per capita disposable income differentiation. Based on the coefficient of variation results, households with labor force members in Qinghai Province are generally concentrated in a certain age group, while in Anhui Province, labor force members are distributed across various age groups. In terms of means, the number of individuals engaged in multiple jobs in the household is higher among interviewed farmers in Qinghai Province compared to Anhui Province, which may be related to the topography and rural development situation in Qinghai Province.

In terms of social support strategies, there are significant differences between interviewed farmers in Anhui Province and Qinghai Province in terms of credit support and relocation policies. Based on the mean results, interviewed farmers in Qinghai Province receive less credit support compared to those in Anhui Province, but there is a higher number of individuals undergoing relocation in Qinghai Province.

In terms of geographical environment, there is a significant difference in terrain fluctuation between Anhui Province and Qinghai Province. This is mainly due to Qinghai Province being located on the first step of China’s terrain, while Anhui Province is situated on the third step. The geographical heterogeneity between the two provinces is significant.

### 3.2. Geodetector calculation results

By inputting the 1,227 questionnaire data from Anhui Province and Qinghai Province into the geographical detector, the output results of single-factor explanatory power and two-factor interaction are obtained (Table 3): The single-factor explanatory power from high to low is *x*_7_ > *x*_16_ > *x*_9_ > *x*_10_ > *x*_11_ > *x*_17_ > *x*_2_ > *x*_12_ > *x*_5_ > *x*_15_ > *x*_14_ > *x*_6_ > *x*_1_ > *x*_8_ > *x*_13_ > *x*_3_ > *x*_4._ Compared to single-factor effects, some two-factor interactions show changes in the q-value. Among the results of two-factor interactions, the differentiation of land management purposes (*x*_7_) has the strongest influence on other factors, with an increased q-value compared to the single-factor effect. The interaction between location distance (*x*_16_) and other factors is second only to the differentiation of land management purposes (*x*_7_). The results indicate that:

1. The willingness of farmers to transfer land is influenced by multiple factors, and the comprehensive results of single-factor detection and two-factor interaction can better explain the factors influencing farmers’ willingness to transfer land use rights.
2. The types of pairwise factor interactions mostly belong to the type of two-factor enhancement, indicating that the willingness of farmers in Anhui Province and Qinghai Province to transfer land is not only influenced by a single factor but is the result of the combined effect of different influencing factors. The interaction effect is greater than the sum of the two factors, creating a “1+1>2” effect.
3. Some factor interactions weaken the willingness of farmers to transfer land, which provides certain references for future land transfer policy formulation.

**Table 3:** Analysis of geographic detector impact factors. (https://github.com/zhangershen/DATE.git).

Influence Factors	Explanatory Power	Influence Factors	Explanatory Power
Age (*x*_1_)	0.168**	Proportion of Wage Income from Employment (*x*_10_)	0.750***
Education Level (*x*_2_)	0.337***	Per Capita Disposable Income (*x*_11_)	0.507***
Health Level (*x*_3_)	0.118***	Minimum Guarantee Policy (*x*_12_)	0.308***
Number of Household Labor Force (*x*_4_)	0.103***	Credit Support (*x*_13_)	0.147**
School-Age Children (*x*_5_)	0.236**	Labor Service Economy (*x*_14_)	0.208***
Cultivated Land Area (*x*_6_)	0.188***	Relocation policy (*x*_15_)	0.211***
Diversification of Cultivated Land Management Objectives (*x*_7_)	0.924***	Location Distance (*x*_16_)	0.857***
Number of Job Categories (*x*_8_)	0.155***	Terrain Fluctuation (*x*_17_)	0.364***
Proportion of Agricultural Operating Income (*x*_9_)	0.855**		

Note: *** and ** represent significance at the 1% and 5% levels, respectively, in the single-factor detection results.

### 3.3. Binary logistic model results

Using Stata 16.0 software, the indicators selected by the geographical detector were standardized using z-scores. The collinearity test was then applied to validate the independent variables. After passing the validation, the final data was input into the Binary Logistic Model to obtain the estimated results of factors influencing farmers’ willingness to transfer land in Qinghai Province and Anhui Province (Table 4).

**Table 4:** The Significance and modes of influence on farmers’ land transfer intentions. (https://github.com/zhangershen/DATE.git).

Endowment Type	Influencing Factors	Anhui Province		Qinghai Province	
		B_1_	Exp(B_1_)	B_2_	Exp(B_2_)
Individual characteristics of the household head	Age (*x*_1_)	1.225***	1.220	-0.245	2.605
	Education Level (*x*_2_)	-2.315***	4.356	-1.655***	2.507
	Health Level (*x*_3_)	3.553***	2.534	2.360***	3.034
Family endowment situation	Number of Household Labor Force (*x*_4_)	-1.192***	6.473	-1.322***	6.212
	School-Age Children (*x*_5_)	-2.072***	3.365	1.212***	1.505
	Cultivated Land Area (*x*_6_)	-1.329***	3.261	-2.172***	3.220
	Diversification of Cultivated Land Management Objectives (*x*_7_)	3.808**	4.885	2.614**	5.224
	Number of Job Categories (*x*_8_)	0.912	3.276	-2.160***	1.188
	Proportion of Agricultural Operating Income (*x*_9_)	-2.629***	1.055	-2.390***	2.027
	Proportion of Wage Income from Employment (*x*_10_)	-0.395	1.946	-2.547***	5.036
	Per Capita Disposable Income (*x*_11_)	0.223	8.693	-0.124	2.667
Social assistance strategies	Minimum Guarantee Policy (*x*_12_)	-0.232	1.184	2.841***	2.116
	Credit Support (*x*_13_)	-0.182	2.723	1.619***	1.514
	Labor Service Economy (*x*_14_)	0.543***	5.434	2.403***	1.028
	Relocation policy (*x*_15_)	1.555*	1.146	2.197*	1.763
Geographic environment	Location Distance (*x*_16_)	-0.462**	1.967	1.582**	2.138
	Terrain Fluctuation (*x*_17_)	-0.324***	1.674	1.146***	1.362
N		712		515	

Note: ***, **, and * indicate significance at the 1%, 5%, and 10% levels, respectively.

Based on the results of the binary Logistic regression model, it was found that there are significant differences in the factors influencing farmers’ willingness to transfer land management rights in the two provinces and their modes of influence among household individual characteristics, household endowments, social assistance strategies, and geographical environment. When other influencing factors are held constant, the change in a single significant independent variable, whether increased or decreased, leads to a change in the dependent variable, determined by the odds ratio Exp(B_1_). In the regression results of factors influencing farmers’ willingness to transfer land management rights in Anhui Province, the age of the household head (*x*_1_), the health status of the household head (*x*_3_), the purpose of land management (*x*_7_), labor economy (*x*_14_), and relocation policy (*x*_15_) are the main promoting factors. On the other hand, the educational level of the household head (*x*_2_), the number of household labor force (*x*_4_), the situation of school-age children (*x*_5_), land area (*x*_6_), the proportion of agricultural operating income (*x*_9_), location distance (*x*_16_), and terrain undulation (*x*_17_) are the main inhibiting factors.

In the regression results of factors influencing the willingness of farmers in Qinghai Province to transfer land management rights, the health status of the household (*x*_3_), the situation of school-age children (*x*_5_), the purpose of land management (*x*_7_), minimum guarantee policy (*x*_12_), credit support (*x*_13_), labor economy (*x*_14_), relocation policy (*x*_15_), location distance (*x*_16_), and terrain undulation (*x*_17_) are the main promoting factors. Meanwhile, the educational level of the household head (*x*_2_), the number of household labor force (*x*_4_), land area (*x*_6_), the number of occupationally differentiated family members (*x*_8_), the proportion of agricultural operating income (*x*_9_), and the proportion of work-related income (*x*_10_) are the main inhibiting factors.

A comprehensive comparative analysis of the binary logistic regression model results for farmers’ land transfer intentions in the two provinces revealed common factors with significant influence, primarily *x*_2_, *x*_3_, *x*_4_, *x*_6_, *x*_7_, *x*_9_, *x*_14_, and *x*_15_. Among the differentiating factors with significant influence were mainly *x*_1_, *x*_5_, *x*_8_, *x*_10_, *x*_12_, *x*_13_, *x*_16_, and *x*_17_.

## 4. Analysis of influencing factors

Based on the results of the influencing factor model for land transfer in Anhui and Qinghai Province (Tables 3 and 4), there are both common and different influencing factors. Therefore, the discussion in this study will focus on the common and different aspects of the land transfer intention of farmers in the two provinces.

### 4.1. Analysis of common influencing factors

#### 4.1.1. Analysis of the common impact effects of household head characteristics on land transfer intentions

The health level of surveyed farmers in Anhui Province and Qinghai Province has a positive effect at the 1% level, indicating that farming households with healthier household heads are more willing to participate in land transfer [70]. China’s urbanization construction has significantly improved the quality of life for both urban and rural residents, but it has also increased the cost of living and education for families. Households with healthy heads generally spend less time and energy caring for elderly family members. This allows for more dual-employment time and opportunities for the workforce, leading to higher returns compared to engaging in agricultural activities. Therefore, such households are more willing to participate in land transfer, effectively enhancing economic income through the value of cultivated land. However, if the household head is ill or physically disabled, the family workforce tends to engage in agricultural or non-agricultural economic activities in nearby areas. As cultivated land serves as a crucial source of stable income, the willingness to participate in land transfer is lower in such cases. Furthermore, during the survey process, it was observed that some households, influenced by the cultural idea of traditional small-scale farming and attachment to their homeland, are reluctant to transfer all of their land even if their labor force is working outside the village. The level of education of household heads in Anhui Province and Qinghai Province has a negative effect at the 1% level, indicating that farmers with higher levels of education have weaker intentions to transfer their land. This could be because farmers with higher levels of education, due to their continuous exposure to education outside the village, are less familiar with land transfer policies and are influenced by the idea of “returning to their roots”, resulting in weaker intentions to transfer their land [29, 34].

#### 4.1.2. Analysis of the common impact effects of household endowment on land transfer intentions

The current cultivated land area of the interviewed households in Anhui Province and Qinghai Province has a negative effect at the 1% significance level, indicating that households with larger land resources are less inclined to participate in land transfer [71]. Among the interviewed households in both provinces, those with cultivated land area higher than the average are primarily engaged in cultivation for meeting food consumption and increasing income, and they are not willing to participate in land transfer. In households with abundant cultivated land resources, the economic income generated from agricultural activities becomes an important source of household income. At the same time, households with limited land resources find that solely relying on agricultural activities does not significantly increase their economic income. Members of such households with labor force are more willing to move away from agricultural activities and engage in higher-paying labor or entrepreneurial activities [72].

The factor of the purpose of land cultivation for the interviewed households in Anhui Province and Qinghai Province has a positive effect at the 5% significance level. This indicates that households still engaged in agricultural activities no longer prioritize consumption but focus more on improving the level of household economic income. Their decisions regarding land transfer are not based on ensuring the basic living conditions of the family but rather on enhancing household income. These households also have a certain economic foundation that allows them to transfer land while assuming minimal risks. Therefore, they hope to transfer their privately-owned land to reduce the time and energy spent on farming. The proportion of agricultural operating income for the interviewed households in Anhui Province and Qinghai Province has a negative effect at the 1% significance level, indicating that households with a higher proportion of agricultural income have a lower willingness to participate in land management and transfer. One possible reason is that households with a higher proportion of agricultural income continue to engage in agricultural production because the income derived from it is higher than the financial gain from land transfer [31]. The research found that households with a higher proportion of income from agricultural activities are mostly engaged in large-scale crop cultivation and have sufficient labor force. They still rely on the value generated by land resources in various aspects of production and livelihood. Therefore, their willingness to participate in land transfer is lower. However, when the value of products produced from cultivated land is lower than the wage income obtained from non-agricultural work, farmers are willing to participate in land management and transfer.

#### 4.1.3. Analysis of the common impact effects of social assistance strategies on land transfer intentions

Labor-intensive economic activities positively influence land transfer intentions for farmers in both Anhui Province and Qinghai Province at a significance level of 1%. This indicates that households participating in organized labor activities organized by the government are more willing to transfer the land use rights of their cultivated land resources. Organized labor output not only provides stable income opportunities for migrant workers but also reduces the risk of market competition failure. For households with migrant workers, the high opportunity cost of labor may lead to a shortage of labor for agricultural activities. This provides an opportunity for household labor to engage in other production activities after moving away from farming. Relocation policies positively influence land transfer intentions at a significance level of 10%, suggesting that households involved in relocation are more willing to transfer their cultivated land resources. After relocation, households are basically separated from their original production and living environment. Returning to their original place of residence for agricultural activities leads to increased input costs. However, improved infrastructure and living conditions in relocation settlements, along with land transfer, can release labor resources from agricultural operations. Households with labor capacity are more willing to seek higher-paying employment and entrepreneurial opportunities. Therefore, they have a higher willingness to transfer land [32, 33].

### 4.2. Analysis of differentiating factors

#### 4.2.1. Analysis of the differential impact effects of household endowment on land transfer intentions

The presence of school-age children has a negative effect, significant at the 1% level, on land transfer willingness among farmers in Anhui Province, while it exhibits a positive effect, significant at the 1% level, among farmers in Qinghai Province. This indicates that in Anhui Province, farmers are unwilling to participate in land transfer because they can obtain income higher than rental fees by operating relatively fertile land, providing sufficient economic support for their school-age children. On the other hand, farmers in Qinghai Province, due to the poor quality of arable land resources and harsh farming environments, have lower land income that cannot meet the education costs beyond living expenses. Therefore, they are more inclined to transfer land and seek higher-income jobs outside to meet their overall family needs. The number of family members engaged in differentiated occupations has a negative effect at the 1% significance level on land transfer willingness for Qinghai Province, but it has no effect on farmers in Anhui Province. This indicates that in Qinghai Province, the more labor force engaged in multiple occupations among family members, the less willing they are to participate in land transfer. With the gradual achievement of agricultural modernization, the time spent by family members on agricultural production and business activities has been reduced. The economic income obtained from the output of cultivated land resources can effectively improve the living standards of the family, weakening the willingness of farmers to participate in land transfer [28, 30, 31]. The proportion of income from wage labor has a negative effect at the 1% significance level on land transfer willingness for Qinghai Province, but it has no effect on farmers in Anhui Province. According to the research data, compared to farmers in Anhui Province, most of the families in Qinghai Province with a high proportion of wage income (over 50%) have more than two laborers. Based on the need for production, consumption, and living expenses, they prefer to increase various sources of income to raise the overall family income rather than participating in land transfer, as the income from agricultural production exceeds the income from land transfer.

#### 4.2.2. Analysis of the differential impact effects of social assistance strategies on land transfer intentions

The minimum livelihood guarantee policy has a positive effect at the 1% significance level on land transfer willingness for surveyed farmers in Qinghai Province, but it has no effect on farmers in Anhui Province. This indicates that in Qinghai Province, the relationship between social security issues and land transfer is closely related to the influence of geographical and economic development conditions on farmers. Farmers who enjoy more social security policies often receive higher economic compensation than the income from cultivated land, thus they are more inclined to participate in land transfer [32, 33]. However, for farmers in Anhui Province, their superior natural conditions, land scale, production and management conditions, crop planting structure, and the quality of farmers themselves weaken the impact of the income effect of the minimum livelihood guarantee policy. Credit support has a positive effect at the 1% significance level on land transfer willingness for farmers in Qinghai Province, but it has no effect on farmers in Anhui Province. This indicates that compared to farmers in Anhui Province, credit support can significantly enhance the production and lifestyle of farmers in Qinghai Province. The use of credit funds can engage in other high-yielding production activities, making farmers with weaker agricultural operating capacity or those who want to change their production methods more eager to participate in land transfer.

#### 4.2.3. Analysis of the differential impact effects of geographic environmental conditions on land transfer intentions

The location distance has a negative effect at the 5% significance level on land transfer willingness for farmers in Anhui Province, while it has a positive effect at the 5% significance level on land transfer willingness for farmers in Qinghai Province. This indicates that for surveyed farmers in Anhui Province, those who live far from the county town have a lower willingness to participate in land transfer. Farmers in relatively remote areas of Anhui Province have limited understanding of land transfer and consider land as their last resort for livelihood, resulting in weaker intentions for land transfer. On the other hand, farmers who live closer to towns are more influenced by urban development and the driving force of urbanization, leading to a higher willingness to participate in land transfer. For surveyed farmers in Qinghai Province, areas closer to the county town have better geographical locations with greater potential for land appreciation, resulting in a lower willingness of farmers’ households to participate in land transfer. The terrain fluctuation has a negative effect at the 1% significance level on land transfer willingness for farmers in Anhui Province, indicating that farmers in low-altitude areas are more willing to participate in land transfer compared to those in high-altitude areas. One possible reason is that farmers in low-altitude areas have better information reception and processing capabilities, and their families may have a stronger awareness of moving away from traditional rural society. The terrain fluctuation has a positive effect at the 1% significance level on land transfer willingness for farmers in Qinghai Province, indicating that farmers in low-altitude areas are less willing to participate in land transfer compared to those in high-altitude areas [34, 37]. This could be due to the poorer land quality in high-altitude areas, making agricultural activities more difficult for farmers and leading them to be more inclined to transfer land.

## 5. Discussion

**Fig 3:**
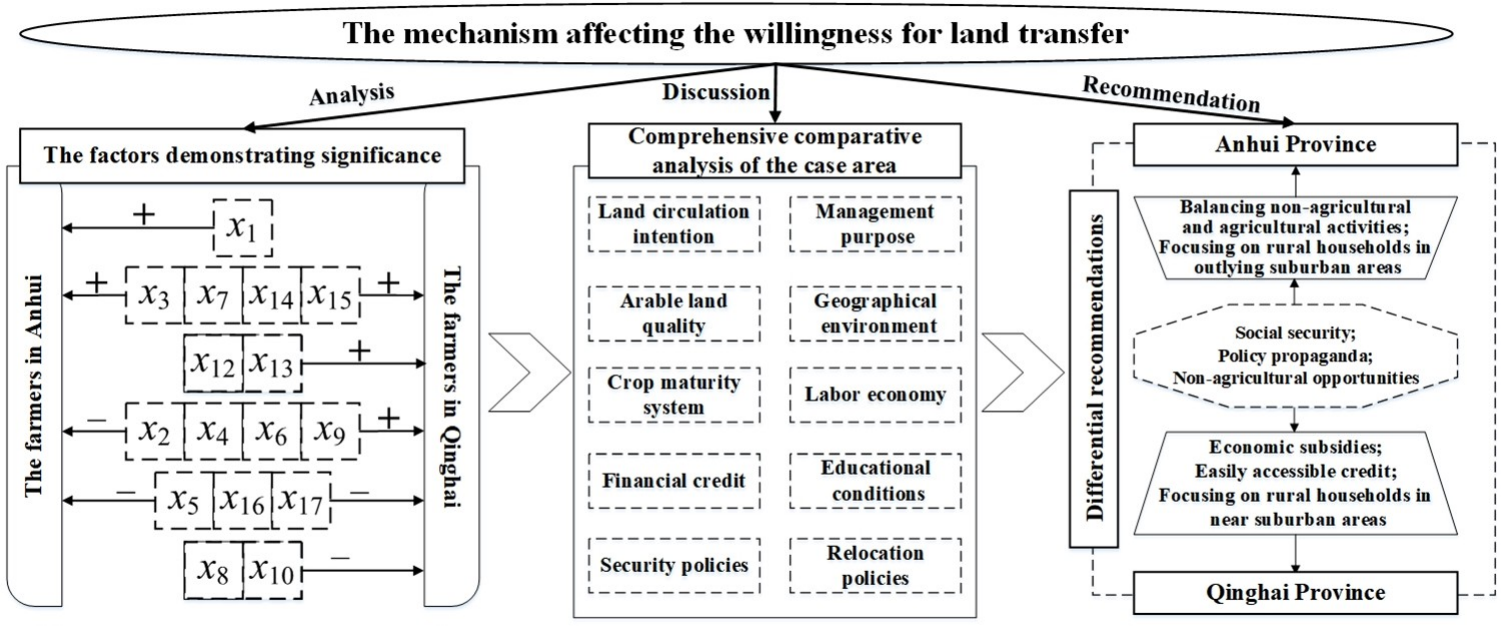
Factor discussion framework.

Through comprehensive on-site investigation of household situations and quantitative analysis results, it was found that there are both commonalities and differences in the factors influencing the willingness to participate in land transfer in Anhui Province and Qinghai Province. Therefore, a framework for discussing these factors is constructed (Fig 3), and relevant recommendations are proposed.

### 5.1. Comprehensive analysis

By conducting statistical analysis of the survey questionnaires on land transfer willingness among farmers in Anhui and Qinghai provinces, it is found that there are certain differences in the willingness to participate in land transfer among households in different geographical areas. An analysis of the driving factors behind land transfer willingness for farmers in both provinces reveals that the purpose of land cultivation and labor economics significantly influence their intentions. This suggests that households with a higher level of participation in labor-intensive economic activities are more inclined to engage in land transfer when land cultivation does not solely satisfy their own consumption needs.

In contrast to the influencing factors of land transfer willingness in rural areas of Anhui Province, the provision of welfare policies becomes a significant factor affecting land transfer among households in Qinghai Province. Land is not only the most important means of production for farmers but also their ultimate security. In rural areas, particularly where social security, especially old-age insurance, is lacking, land plays a vital role in providing security for the elderly [15, 30]. However, as rural social security, especially rural pension security, improves, the ultimate security function of land management will correspondingly decrease. Low-income farmers who receive national minimum subsistence allowances and enjoy the “five-guarantee” policy (the five gurantee measures of living security for eligible rural dependents, including food, clothing, safety, medical care and burial), when faced with the challenge of having land resources but lacking the ability or insufficient capacity for cultivation, may choose to transfer their land to increase income and alleviate their living pressures [31, 36]. Furthermore, in terms of social assistance, the impact of national basic security policies and relocation policies on farmers’ willingness to participate in land transfer is more pronounced in Qinghai Province [32, 33]. In comparison, farmers in Anhui Province who receive minimum livelihood guarantees have higher-quality land resources than those in Qinghai Province, and the mechanism of two crop harvests a year can satisfy their own consumption needs. As a result, their willingness to participate in land transfer is weaker. Compared to Anhui Province, Qinghai Province has relocated households from the harsh high-altitude environment to more suitable areas for living. Due to the distance limitation between production and residence, they are more willing to transfer the land in their original residential area.

In Qinghai Province, households with more arable land resources mainly focus on pasture and highland barley cultivation, facilitates their achievement of agricultural modernization and their engagement in large-scale planting and breeding activities. Therefore, their willingness to transfer land is not strong. On the other hand, families with less arable land resources and greater reliance on agricultural output are less willing to continue agricultural activities in the future. In households with a higher degree of occupational differentiation, the increasing number of family members engaged in multiple occupations indicates a gradual transition from purely farming households to households engaged in multiple occupations. As a result, the significance of land resource returns within the family gradually decreases. However, farmers themselves have an emotional attachment to the land, which results in a lower willingness to transfer it.

### 5.2. Recommendations

#### 5.2.1 Addressing common factors affecting farmers’ participation in land transfer activities in both provinces

1. Provide targeted social security services to reduce farmers’ reliance on operational income from land. For farmers who consider crop income as their pension security, the government and relevant departments should strengthen the implementation of rural social security policies. This includes offering more comprehensive medical and elderly care services to encourage households with healthier heads to reduce excessive dependence on agricultural operational income and enhance their willingness to participate in land transfer. For migrants benefiting from relocation policies, diverse livelihood recovery measures should be implemented to facilitate integration into the community. This can involve improving infrastructure, providing educational assistance, offering employment information, and conducting skills training.
2. Strengthen the publicity of land transfer policies to enhance the enthusiasm of participants. Tailor education and awareness campaigns about land transfer policies to farmers with different levels of education to help them better understand the pros and cons of participating in land transfer activities. Particularly for farmers with a higher degree of dual employment, reinforce support for their labor output and organizational work. Provide more information on job opportunities and training, encouragingparticipation in land transfer to improve regional land utilization rates.
3. Increase non-agricultural income opportunities for farmers and optimize the structure of household income. Provide early non-agricultural business and employment guidance services to farmers with large areas of cultivated land and a high proportion of agricultural operating income. Encourage them to explore other economic income growth opportunities. Ensure sustainable livelihoods post-land transfer by enhancing household economic diversity to reduce excessive dependence on agriculture. Additionally, for diverse households, offer more support measures to enhance household economic diversity.

#### 5.2.2 Addressing differentiating factors affecting farmers’ participation in land transfer activities in both provinces

For farmers in Qinghai Province, accessible credit support can be provided to those in need. This assistance aims to help labor forces engage in higher-income production activities. Additionally, families with school-age children can be offered education subsidies, and economic compensation can be increased for households benefiting from the minimum guarantee policy. Concerning farmers in Anhui Province, support can be extended to households with a higher degree of dual employment to balance the relationship between non-agricultural economic activities and agricultural activities. This can involve releasing labor occupied by agricultural operations and strengthening the radiative effects of land transfer policies in suburban areas. These recommendations should be tailored to the local context and policies, with continuous monitoring and adjustments to ensure effectiveness. Moreover, collaboration among the government, research institutions, and rural communities is essential to collectively promote land transfer for the sustainable development of rural economies.

In summary, the Chinese government should comprehensively advance land transfer policies to enhance the efficiency of cultivated land utilization. This can be achieved by promoting participation in land transfer activities through means such as educational campaigns, improving social security policies, providing training and financial support, and enhancing land quality. However, encouraging unilateral participation in land transfer by farmers alone is unsustainable. The government should play a role in information dissemination and consultation services, fostering a diverse range of entities to create a positive land transfer market environment. Additionally, strengthening supervision, legal safeguards, and boosting farmers’ confidence are essential.

For instance, the government can establish more effective information dissemination and consultation services, providing farmers with information on land transfer policies, market opportunities, and agricultural technologies to assist them in making informed decisions. Encouraging and supporting the involvement of diverse entities in constructing the land transfer market will provide more transaction convenience and legal protection, reducing risks faced by various participants. Furthermore, in policy formulation, the government can customize land transfer policies based on the characteristics of farmers in different regions to meet their diverse needs and circumstances. Rigorous oversight of land transfer contracts should be implemented to ensure the protection of farmers’ rights and reduce the risk of contract disputes.

### 5.3. Limitations

This study primarily used a mix of qualitative and quantitative methods to investigate the factors influencing farmers’ land transfer decisions during a specific period, but did not explore the dynamic changes in land transfer decision-making behavior. Therefore, future research can employ long-term field surveys and conduct periodic in-depth interviews to further explore the dynamic factors behind farmers’ land transfer decisions. Additionally, future studies should focus on the willingness to transfer land among different regions and different types of farmers (such as low-income farmers, high-income farmers, etc.) to delve deeper into the factors influencing the willingness of different types of farmers to transfer land.

## 6. Conclusion

Based on field survey data, government statistics, and terrain data, the study employed Geographical Detector and Binary Logistic Model to explore the common and differential factors influencing farmers’ willingness to transfer land management rights in different topographic regions. The following conclusions are drawn:

1. By comparing and analyzing the mean, standard deviation, and coefficient of variation of 17 indicators in 4 dimensions of the two-dimensional data from Anhui and Qinghai provinces, it is found that the characteristics of individual household heads, household endowments, social assistance strategies, and geographical environment all show significant differences and substantial numerical variations. This indicates that there are significant variations in the fundamental characteristics and living conditions of farmers in both provinces.
2. There are significant differences in the explanatory power of single-factor influences on farmers’ land transfer willingness, and various types of two-factor interactions exist. The order of single-factor explanatory power from high to low is *x*_7_ > *x*_16_ > *x*_9_ > *x*10 > *x*_11_ > *x*_17_ > *x*_2_ > *x*_12_ > *x*_5_ > *x*_15_ > *x*_14_ > *x*_6_ > *x*_1_ > *x*_8_ > x_13_ > x_3_ > x_4_, all significant at the 1% level. The geographical detector model reveals the enhancing or weakening effects on land transfer willingness due to the interaction between any two factors. Combined with the results of Binary Logistic Model, the fundamental reason lies in the differences in the ways different independent variables affect the dependent variable.
3. There are commonalities in the modes of action of factors influencing the willingness of farmers in different geographical areas to participate in land transfer. Factors such as the educational level of the household head, the health level of the household head, the number of family laborers, cultivated land area, differentiation of land cultivation purposes, the proportion of agricultural operating income, labor economics, and relocation policies exhibit the same mode of influence on land transfer willingness for farmers in both provinces.
4. There are differences in the modes of action of factors influencing the willingness of farmers in Anhui and Qinghai provinces to participate in land transfer. The differentiating factors with significant effects mainly include the age of the household head, the number of school-age children, the number of family members with occupational differentiation, the proportion of income from off-farm work, minimum guarantee policies, credit support, location distance, and terrain fluctuation. Among them, the age of the household head significantly promotes land transfer willingness among farmers in Anhui Province but is not significant in Qinghai Province. The number of family members with occupational differentiation, the proportion of income from off-farm work minimum guarantee policies, and credit support are significant factors influencing land transfer willingness among farmers in Qinghai Province but not significant in Anhui Province. In addition, The presence of school-age children, location distance, and terrain fluctuation significantly hinder land transfer willingness among farmers in Anhui Province but significantly promote it among farmers in Qinghai Province.
